# A chronological history of heart valve prostheses to offer perspectives of their limitations

**DOI:** 10.3389/fbioe.2025.1533421

**Published:** 2025-02-14

**Authors:** Raquel Ahnert Aguiar Evangelista, Ana Luiza Resende Pires, Breno Valentim Nogueira

**Affiliations:** ^1^ Rede Nordeste de Biotecnologia (RENORBIO), Federal University of Espírito Santo (UFES), Vitória, Brazil; ^2^ Graduate Program in Biotechnology, Federal University of Espírito Santo. Av. Marechal Campos, Vitória, Brazil

**Keywords:** heart valve diseases, valve repair, valve replacement, mechanical valves, bioprosthetic valves, biomaterials, tissue engineering

## Abstract

Prosthetic heart valves (PHV) have been studied for around 70 years. They are the best alternative to save the life of patients with cardiac valve diseases. However, current PHVs may still cause significant disadvantages to patients. In general, native heart valves show complex structures and reproducing their functions challenges scientists. Valve repair and replacement are the options to heal heart valve diseases (VHDs), such as stenosis and regurgitation, which show high morbidity and mortality worldwide. Valve repair contributes to the performance of cardiac cycles. However, it fails to restore valve anatomy to its normal condition. On the other hand, replacement is the only alternative to treat valve degeneration. It may do so by mechanical or bioprosthetic valves. Although prostheses may restructure patients’ cardiac cycle, both prostheses may show limitations and potential disadvantages, such as mechanical valves causing thrombogenicity or bioprosthetic valves, calcification. Thus, prostheses require constant improvements to remedy these limitations. Although the design of mechanical valve structures has improved, their raw materials cause great disadvantages, and alternatives for this problem remain scarce. Cardiac valve tissue engineering emerged 30 years ago and has improved over time, e.g., xenografts and fabricated heart valves serving as scaffolds for cell seeding. Thus, this review describes cardiac valve substitutes, starting with the history of valvular prosthesis transplants and ending with some perspectives to alleviate the limitations of artificial valves.

## Graphical Abstract

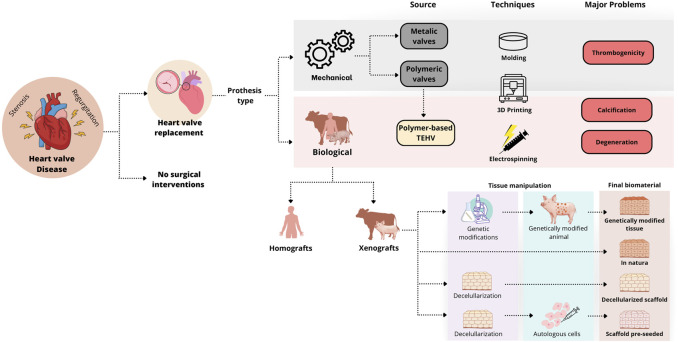

## 1 Introduction

Hearts are valvular and muscular pumps of the size of a fist and weigh approximately 300 g in healthy adults. Normal physiologic hearts pump about 7000 L of blood each day, and their one-way blood flow is due to four valves, which prevent blood from returning (Weinhaus, 2015; Whitaker, 2014). According to the World Health Organization (WHO), cardiovascular diseases (CVDs) are the greatest cause of death globally. Moreover, the surgical cost for some disorders may be high, such as valve repair and replacement. Statistically, heart valve diseases (VHDs) affect approximately 2.5% of the general population when adjusted for age and sex distribution. The prevalence increases significantly with age, ranging from 0.7% in individuals aged 18–44 years to 13.3% in those aged 75 years and older. This highlights the disproportionate burden of valve disease in elderly populations, which necessitates increased focus on age-related diagnostic and therapeutic strategies (Nkomo et al., 2006). In general, estimates suggest that the global proportion of people aged above 60 years will reach 21% until 2050; thus, the annual number of valve transplants is also expected to increase, reaching 850,000 implants (Fioretta et al., 2021; Yacoub and Takkenberg, 2005).

Abnormal heart valve structures and functions, such as stenosis and regurgitation, can drastically affect patients’ cardiac cycle (Maganti et al., 2010; Schoen, 2012). Treatment includes repairing or replacing diseased valves to restore patients to a normal cardiac cycle. Repairing valves reconstructs patients’ heart function but is unable to maintain the normal anatomy of heart valves, whereas replacement involves implanting mechanical or bioprosthetic valves so patients’ hearts can function close to the normal (Cheung et al., 2015; Rimmer et al., 2019).

Valve replacement has been carried out since the 1950s. This practice is considered the most effective alternative for several valvular heart diseases, and, nowadays, 300,000 valves are replaced each year. But the heart valve prosthetics are not perfect; studies have constantly sought to improve the current models (Dai et al., 2019; Dasi et al., 2009). Mechanical valves have a durable structure, and biological valves show a structure and anatomy that resembles the native valve (Fiedler and Tolis, 2018; Head et al., 2017). However, research still finds limitations in both mechanical and bioprosthetic valves. The biomaterials used to produce mechanical valves may cause thrombogenicity and thus patients’ dependence on anticoagulants, whereas bioprosthetic valves may deteriorate due to calcification (Head et al., 2017). Despite the limitations, valvular prosthesis implantation is the better alternative to improve patients’ quality of life. This explains the growth of its market each year, which reached US$4.8 billion in 2017, and estimates suggested an increase to US$8.9 billion by 2022 (Sotiri et al., 2019), which in fact occurred in 2023. Estimates of the Compound Annual Growth Rate (CAGR) range from 7.5% to 12.9% per year, with the market size projected to reach around US$30 billion by 2035.

Given this scenario, perspectives to solve the limitations of prosthetic heart valves require further research and improvements. Thus, studies must seek biomaterials for mechanical valves that avoid causing thrombogenicity (Sotiri et al., 2019) and alternatives to prevent the calcification of bioprosthetic valves. Tissue engineering applied to bioprosthetic and fabricated heart valves currently offers a promising panorama for valve efficiency and longevity by, e.g., enabling artificial valves to release bioactive compounds to better adapt to patients’ bodies without long-term complications.

Given the advancements and challenges in the field of cardiac valve prostheses, this review aims to provide a comprehensive analysis of the topic. We begin by exploring the anatomy and function of natural heart valves, followed by a discussion on the conditions requiring valve replacement and the available treatment options. Next, we examine the historical evolution of prosthetic valves, highlighting key technological milestones. To deepen the understanding of prosthetic performance, we present critical concepts in biomechanics and hemodynamics. Furthermore, we discuss the types of prosthetic valves currently in use, their limitations, and the challenges they pose. Finally, we explore future perspectives, including biofabricated valves, hybrid designs, and innovative technologies that hold promise for next-generation valve replacement and repair.

## 2 Natural heart valves

### 2.1 Heart valves structure

The unidirectional blood flow, through the heart chambers for the body’s circulation, is ensured by four heart valves (HV), two atrioventricular (tricuspid and mitral) and two semilunar (pulmonary and aortic) valves (Lodhia and Evans, 2018). Chordae tendineae anchor atrioventricular valves, whereas a fibrous skeleton within the aorta or pulmonary root fixates the semilunar valves (Khang et al., 2018). Their matrices have a thin membranous formation called cusps or leaflets, which originate from a substantial organization of collagen, elastin, extracellular proteins, and cells, providing resistance to high blood flows and pressures (Jana et al., 2014). To provide a comprehensive comparison, the key features of these valves are summarized in Table 1.

**TABLE 1:** Comparison of the anatomical location, structure, function, and common pathologies of the four human heart valves (Hinton and Yutzey, 2011).

Valve	Location	Structure	Function	Key pathologies
Aortic	Between the left ventricle and aorta	Three semilunar cusps; high elasticity and resistance	Allows blood flow into the aorta during systole; prevents regurgitation during diastole	Aortic stenosis; aortic regurgitation
Mitral	Between the left atrium and left ventricle	Two leaflets (anterior and posterior) attached to papillary muscles via chordae tendineae	Ensures unidirectional flow from the atrium to the ventricle; prevents backflow	Mitral prolapse; mitral regurgitation; mitral stenosis
Tricuspid	Between the right atrium and right ventricle	Three leaflets supported by chordae tendineae and papillary muscles	Facilitates flow from the atrium to the ventricle; prevents regurgitation during systole	Tricuspid regurgitation; tricuspid stenosis
Pulmonary	Between the right ventricle and pulmonary artery	Three semilunar cusps; lacks chordae tendineae	Permits blood flow into the pulmonary artery during systole; prevents backflow during diastole	Pulmonary stenosis; pulmonary regurgitation

Mitral Valve (MV) and Tricuspid Valve (TV) chordae tendineae constitute a complex web of chordae with collagen-dense structures, which work due to the aid of the papillary muscle. The support to MV and TV is only possible due to the synergism of chordae tendineae and the papillary muscle (Khalighi et al., 2017; Meschini et al., 2018). The cusps of Aortic Valve (AV) are named according to their respective coronary ostia, right coronary, left coronary and non-coronary leaflets. The integrity of these structures ensures normal flow through the coronary, as during diastole, the closure of the leaflets interrupts the blood flow and allows it to flow through the ostia (Schoen, 2012; Lodhia and Evans, 2018).

The complex general structure of HV involves the synergy between matrices, cells, cytokines, and growth factors. AV and PV wall roots have circular-shaped structures and are between 10 and 50-fold stiffer than leaflets. Root diameter is indispensable for the ideal function of these tissues and their dimensions vary according to cardiac cycles. Their structure composition generally resembles that of blood vessels. Thus, these valve parts consist of the intima (basal lamina with endothelial cells), medial (elastin fibers with myoblasts), and adventitial (collagen with fibroblasts) layers (Cheung et al., 2015; Loukas et al., 2014). Knowledge of the characteristics of native cardiac valves is essential to efficiently analyze prostheses.

Among the heart valves, the aortic heart valve (AHV) is the most studied, due to the higher incidence of pathologies and need for transplantation involving this structure (Rabkin-Aikawa et al., 2024). It`s leaflet is a flexible and elastic structure with a central axis of dense fibrous connective tissue, composed mainly of collagen fibers. The total percentage of collagen is approximately 50% by dry weight, with type I collagen predominating (74%), followed by elastin (10%–13%) and glycosaminoglycans (20%) (Rabkin-Aikawa et al., 2024; Bashey et al., 1967; Taylor, 2007; Büttner et al., 2021; Butcher et al., 2008). Histologically, three layers can be identified in the leaflets: ventricularis, spongiosa and fibrosa. Each layer has a specific majority composition, which is directly related to its physical properties (Büttner et al., 2021; Butcher et al., 2008; Eckert et al., 2013).

**FIGURE 1:**
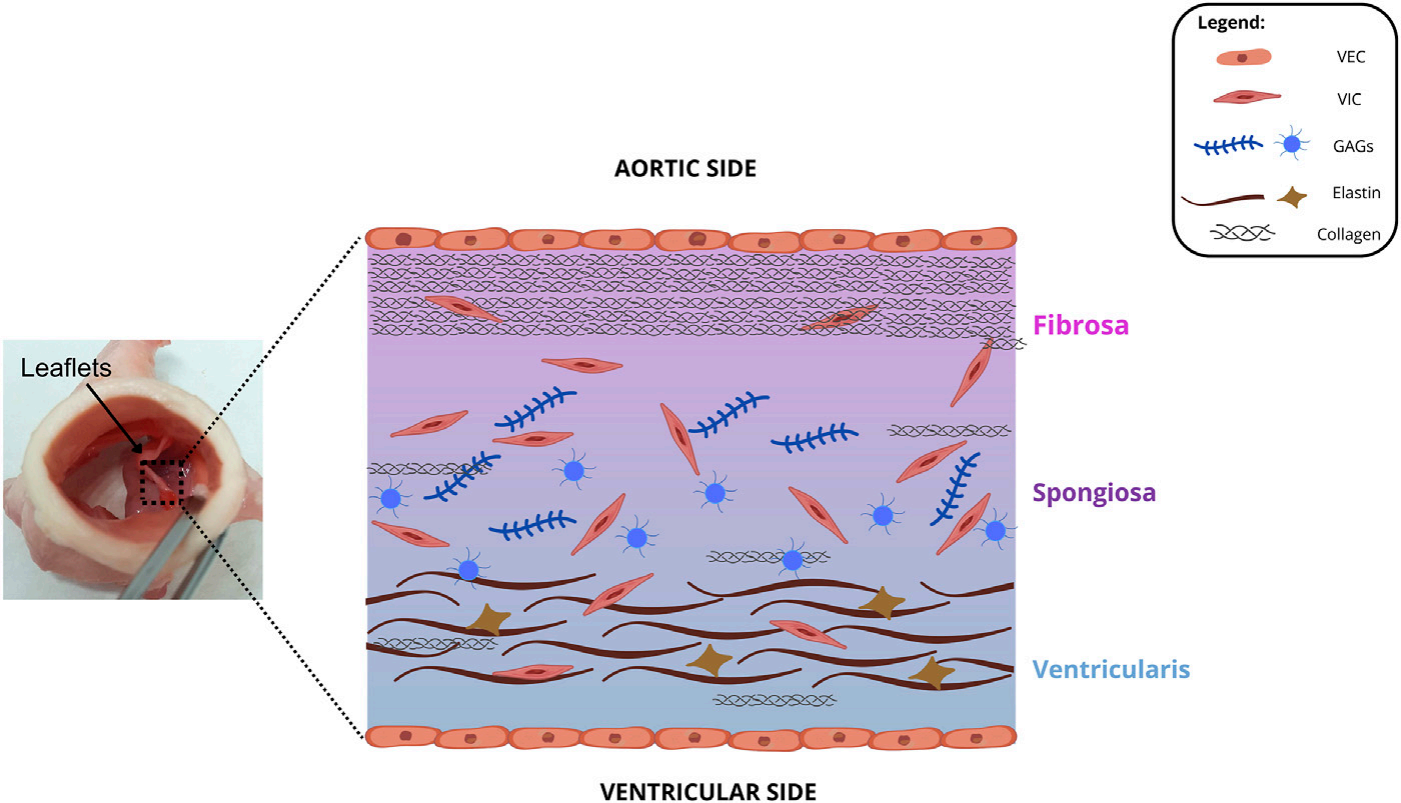
Cross-sectional illustration of the fibrosa, spongiosa, and ventricularis layers. GAGs: glycosaminoglycan, VEC: valvular endothelial cell, VIC: valvular interstitial cell.

The fibrosa is predominantly composed by collagen fibers, which provide mechanical and tensile strength. Elastin fibers have contractile characteristics and can be easily stretched and contracted. As they are the main component of the ventricularis layer, they provide elasticity to the tissue (Zhang et al., 2015). The spongiosa is located between fibrosa and ventricularis and is predominantly composed by glycosaminoglycans, which promote high hydration for the tissue, creating a favorable microenvironment for cellular interactions and communications, in addition to providing resistance to compression forces (Taylor, 2007; Büttner et al., 2021; Eckert et al., 2013; Dainese et al., 2013). Figure 1 shows the layer morphology of aortic valves.

There are predominantly 2 cell types in AHV: valvular endothelial cells (VECs) and interstitial cells (VICs) (Büttner et al., 2021; Zhang et al., 2015; Chester, 2005; Ovalle and Nahirney, 2008; Chester and Grande-Allen, 2020). A single layer of valvular endothelial cells (VECs) sheaths the surface of the leaflets in all four valves and aligns themselves with the orientation circumferential collagen. VECs aim to protect heart valves from structural and functional abnormalities and regulate inflammations, thrombosis, vascular tone, and remodeling (Jana et al., 2016; Sacks and Yoganathan, 2007). Some studies suggest that these cells also act as a possible source of VICs, since their transdifferentiation was possible by using ɑ-smooth muscle actin (SMA) as a stimulus (Büttner et al., 2021; Paranya et al., 2001; Rattazzi and Pauletto, 2015). The transdifferentiation process to myofibroblasts appears to precede the acquisition of osteoblastic characteristics, which may be related to the development of sclerosis and leaf calcification (Büttner et al., 2021; Hjortnaes et al., 2015).

Leaflets also contain valvular interstitial cells (VICs), that can be subdivided into three phenotypes: fibroblastic, myofibroblastic, and osteoblastic. Fibroblastic VICs are predominantly found in mature AHVs. This phenotype has been associated with the maintenance of valve structure and function, while myofibroblastic VICs are closely involved in tissue repair and remodeling (Chester and Grande-Allen, 2020). When activated, the latter express ɑ-smooth muscle actin (SMA), a molecule involved in the migration process, in addition to increasing stiffness and basal tone in porcine aortic valve leaflets (Merryman et al., 2006a). VICs aim to set the leaflet microstructural (glycosaminoglycans, collagen, and elastin) integrity by synthesizing proteins, degrading enzymes, and producing a large number of extracellular matrices with these microstructural components. Additionally, healthy valves have inactive VICs, whereas diseased valves, active VICs, which may enhance calcification (Jana et al., 2016; Sacks and Yoganathan, 2007; Benton et al., 2008; Jana and Lerman, 2019).

### 2.2 Heart valves function

The HV demonstrates a remarkable synergy between their structural design and functional roles, which is essential for maintaining efficient hemodynamic flow. These valves are not only gatekeepers of unidirectional blood flow but also critical modulators of cardiac workload, adapting to the diverse pressures and flow rates across the heart. The structural complexity of the valves, from the atrioventricular valves’ reliance on chordae tendineae to the semilunar valves’ intrinsic elasticity, underscores their evolutionary optimization. Moreover, these adaptations pose significant challenges in replicating native valve performance in prosthetic designs (Hinton and Yutzey, 2011).

Despite the differences in structure and location between the atrioventricular valves and the pulmonary and aortic valves, their primary common function is to facilitate blood flow through the heart chambers while preventing its backflow (as shown in Table 1). The functioning of heart valves occurs during cardiac cycles. In the diastole phase, the atrioventricular valves open their leaflets, allowing blood to fill the heart chambers, while the pulmonary and aortic valves remain closed. When systole begins the leaflets of the MV and TV closes preventing blood backflow to the atria. In the meantime, the pulmonary and aortic valves remain open as blood flows, pushing their leaflets against the walls (Schoen, 2012; Hinton and Yutzey, 2011).

Collagen and elastin fiber orientation significantly influences the mechanical properties of valves since their anisotropy stems from their alignment (Courtney et al., 2006), thus showing distinct stress-strain responses in their circumferential and radial orientations (Weinberg and Kaazempur-Mofrad, 2005). In general, the complex architecture of heart valves promotes both stronger and stiffer leaflet and wall tissues in the circumferential orientation than in radial or axial orientation. This may be explained by the extremely complex multi-axial stress regime to which cardiac valves are subjected, i.e., bending and elongation with cyclic loading (Oveissi et al., 2020). Their complex architecture hinders the reproduction of the mechanical characteristics of native valves (Li et al., 2019). The literature only shows data for the mechanical properties of human aortic (AV) and pulmonary valves (PV), whereas that for human tricuspid (TV) and mitral valves (MVs) remains scarce (Table 2). This is due to AV and PV requiring more medical interventions than MV and TV (Sacks and Yoganathan, 2007). The literature shows that cardiac diseases affect the mechanical properties of cadaveric heart valves and few studies on the mechanical properties of cardiac valves use healthy human hearts. Research has also found that age directly influences the mechanical properties of heart valves since, at the age of 65 years or above, studies have found reduced leaflet stiffness and extensibility (Pham et al., 2017). The AV resistance has been attributed to several factors, including its composition and the intrinsic extracellular matrix (ECM) ability to self-healing, which are related to the cells residents in the tissue (Büttner et al., 2021; Merryman et al., 2006a; Merryman et al., 2006b).

**TABLE 2:** Mechanical properties of human heart valves under circumferential and radial loading conditions. EM: Elastic modulus, TS: tensile strength, EB: elongation at break.

Valve	Mechanical properties						References
	Circumferential			Radial			
	EM (MPa)	TS (MPa)	EB (%)	EM (MPa)	TS (MPa)	EB (%)	
AV	10 to 15	1.74	18	1.98 to 2.32	0.32	24	Jana et al. (2014), Pham et al. (2017), Stradins et al. (2004)
PV	6 to 16	2.78	19	1.32 to 2.17	0.29	30	Pham et al. (2017), Stradins et al. (2004)
MV	8.43 *	-	-	3.65 *	-	-	Pham et al. (2017)
TV	6.33	-	-	2.70	-	-	Pham et al. (2017)

(*) average between anterior and posterior mitral leaflets.

(−) data not found.

## 3 Conditions requiring valve replacement and treatments

### 3.1 Common valve diseases

Any disorder disturbing cardiac physiology requires specific treatment to restore patients’ cardiac cycles. The literature currently considers VHDs a cardiac epidemic due to their high morbidity and mortality worldwide (Petrou and Shah, 2018; Zhu and Grande-Allen, 2018). The structural and functional alterations to damaged valves may be congenital, acquired, or both, and causes may involve aged, calcific, or rheumatic valves, prolapse valve, and others (Schoen, 2012). In general, VHDs comprise defects in one or more valves, causing stenosis and regurgitation, among others (Maganti et al., 2010).

#### 3.1.1 Aortic stenosis (AS)

Stenosis narrows and compresses orifices, restricting flow, imprisoning blood, and inducing pressure in the anterior chamber, a common disease that may cause cardiovascular morbidity and mortality. Its diagnosis starts with physical examinations and referral to echocardiography, the primary non-invasive imaging method to estimate its severity (Maganti et al., 2010; Baumgartner et al., 2009). Although stenosis can affect all valves, it is most prevalent in aortic valves (present in 2% of individuals aged above 65 years), especially after hypertension and coronary artery disease (Maganti et al., 2010; Schoen, 2012).

Aortic stenosis (AS) may be asymptomatic for years while it gradually worsens without patients’ awareness. Obstruction forces the left ventricle to adapt, increasing its wall thickness without changing its size and, despite the pressure gradient, the left ventricle systole and cardiac output are normal (Maganti et al., 2010; Tastet et al., 2019). In general, the pathologies causing AS include calcific degeneration, congenital bi- or unicuspid disease, and post-inflammatory VHDs, such as rheumatic disease (Fishbein and Fishbein, 2019). However, clinical practice has no pharmacologic options to treat or reduce stenosis, rendering aortic valve replacement its only solution (Petrou and Shah, 2018).

#### 3.1.2 Mitral regurgitation (MR)

Regurgitation is the inability of valves to close properly, leading to decreased heart efficiency and inadequate blood pump as it returns to the superior chamber, reducing blood flow and stressing the heart. Regurgitation in aortic and pulmonary valves may occur with root dilatation, which may hinder the adequate movement of its cusps, promoting blood return (Schoen, 2012). However, other mechanisms may cause regurgitation, such as cusp prolapse, retraction by a scar, and perforation. Thus, regurgitation in AV and PV may begin with abnormal leaflets, roots, or both (Maganti et al., 2010; Fishbein and Fishbein, 2019). As with stenosis, regurgitation may be asymptomatic for years, and the only treatment is replacing injured valves. Regurgitation is most prevalent in the mitral valve, preceding AS only in VHD frequency (Petrou and Shah, 2018; Tabata et al., 2019).

Studies have found two types of mitral regurgitation (MR): functional and primary. Functional MR occurs due to pathological myocardial remodeling, deforming it and hindering its closure. Primary MR degenerates leaflets (myxomatous degeneration) due to collagen destruction and glycosaminoglycan deposition in its extracellular matrix. Primary MR causes valve weakness, prolapse, and regurgitation (Zhu and Grande-Allen, 2018). The surgical repair of MV is only applicable for low-to-intermediate risk patients due to the complexity of the MV anatomy. The alternative for patients with high surgical risk is the minimally invasive transcatheter technology, which we explain below (Tabata et al., 2019).

VHD may also include multiple valvular heart disease, which can simultaneously cause stenosis and regurgitation in one or more cardiac valves. Valve replacement constitutes the treatment for its high mortality, and research recommends the same type of prosthesis in both damaged valves (whether bioprosthetic or mechanical) (Venneri et al., 2019). Unger et al. (Unger et al., 2019) reported that a combined aortic and mitral regurgitation remains an understudied multiple valvular heart disease. It may distinctly affect both valves, e.g., both lesions may be severe or one of them, severe and the other, moderate.

### 3.2 Indications for valve replacement

According to the updated 2024 ACC/AHA Guidelines for Valvular Heart Disease (Hussain and Huerter, 2024), valvular disease is classified into four stages: A (at risk), B (progressive), C (severe asymptomatic), and D (severe symptomatic). Initial evaluation includes medical history, physical examination, electrocardiogram, echocardiogram, and chest X-ray. For stage D cases, therapeutic decisions, including surgical treatment, are based on detailed tests and risk assessment tools to optimize intervention outcomes.

### 3.3 Treatment options

VHD treatments begin with a functional and anatomic diagnosis and analysis of patients’ clinical history. Evaluating its etiology and the presence of symptoms is essential to guide the ideal treatment, which may include surgical repair or replacement or transcatheterism. Medication only alleviates symptoms and fails to cure these diseases (Baumgartner et al., 2017; Nishimura et al., 2019).

#### 3.3.1 Valve repair

Valve repair is the option to restore the normal function of valves, rather than their normal anatomy. This surgical option remains rare, such as in patients with a high degree of cardiac disease (insufficiency or regurgitation), under surgical risk of operation and/or thromboembolism, and children. However, surgical results show significant mortality (Cheung et al., 2015; Rimmer et al., 2019; Madesis et al., 2014).

#### 3.3.2 Valve replacement

Studies have found a higher predominance of valve replacement (VR) to treat VHD, which can occur by open-heart surgery or transcatheterism. VR involves implanting mechanical or biological valves since the choice directly depends on the risk of anticoagulation, thromboembolism, valve deterioration, and especially patients’ lifestyle and preferences (Cheung et al., 2015; Baumgartner et al., 2017; Vahanian et al., 2021).

**FIGURE 2:**
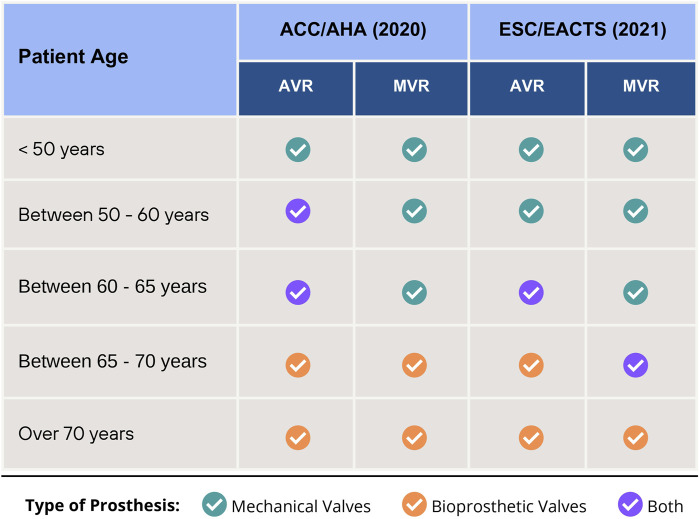
Recommendations for choice of type valve prosthesis for Aortic Valve Replacement (AVR) and Mitral Valve Replacement (MVR), according to 2020 ACC/AHA and 2021 ESC/EACTS Guidelines.

The American College of Cardiology/American Heart Association (ACC/AHA) (Committee Members et al., 2020) and the European Society of Cardiology/European Association for Cardiothoracic Surgery (ESC/EACTS) (Vahanian et al., 2021) offer guidelines on the correct selection of prosthetic valves but differ in recommending aortic valve replacements (AVR) for patients aged 50–60 years and mitral valve replacement (MVR) for those aged 65–70 years. Regarding AVR, the current ESC (Vahanian et al., 2021) guideline recommends a mechanical prosthesis for patients aged <60 years since, for those between 60 and 65 years, mechanical or bioprosthetic valves are acceptable, whereas the ACC recommends both valves for patients aged 50–65 years Figure 2 summarizes the recommendations from each guideline.

Some publications oppose the ACC guidelines. For example, Traxler et al. (Traxler et al., 2021), after comparing the choice between mechanical and bioprosthetic valves in Austrian patients aged 50–65 years, found a greater long-term survival in patients who received mechanical prostheses. The authors reported that patients with bioprostheses show higher reoperation and myocardial infarction risks. Glaser et al. (Glaser et al., 2015) studied patients aged 50–69 years with primary isolated AVR and found that mechanical valve implantation showed higher long-term survival, whereas those aged 50–59 years with bioprosthetic AVRs had a significant association with long-term all-cause mortality.

Despite these results, the choice of prosthetic valves for older patients remains controversial. Another study (Chiang et al., 2014) confirmed that bioprosthetic valves may be used in patients aged 50–69 years, corroborating the ACC guideline and opposing the ESC one for people aged 50–60 years. In contrast to both, Okamoto et al. (2016) retrospective study showed that mechanical valves were acceptable for older patients (i.e., aged >75 years) but due to the possibility of bleeding or thromboembolism, bioprosthetic valves are more commonly used. Thus, the clinical use of mechanical and bioprosthetic valves has shown contradictory results, requiring further studies to aid the correct choice of valves to increase patients’ survival.

As mentioned, the Ross procedure also offers an alternative, a complex operation involving patients’ own healthy pulmonary valves to replace diseased ones (especially aortic valves). In this case, a prosthetic valve is placed in the pulmonary position. The procedure includes a two-valve operation in which both dysfunctional valves fail to perform properly (Cheung et al., 2015; David et al., 2014).

## 4 History of prosthetic heart valves

**FIGURE 3:**
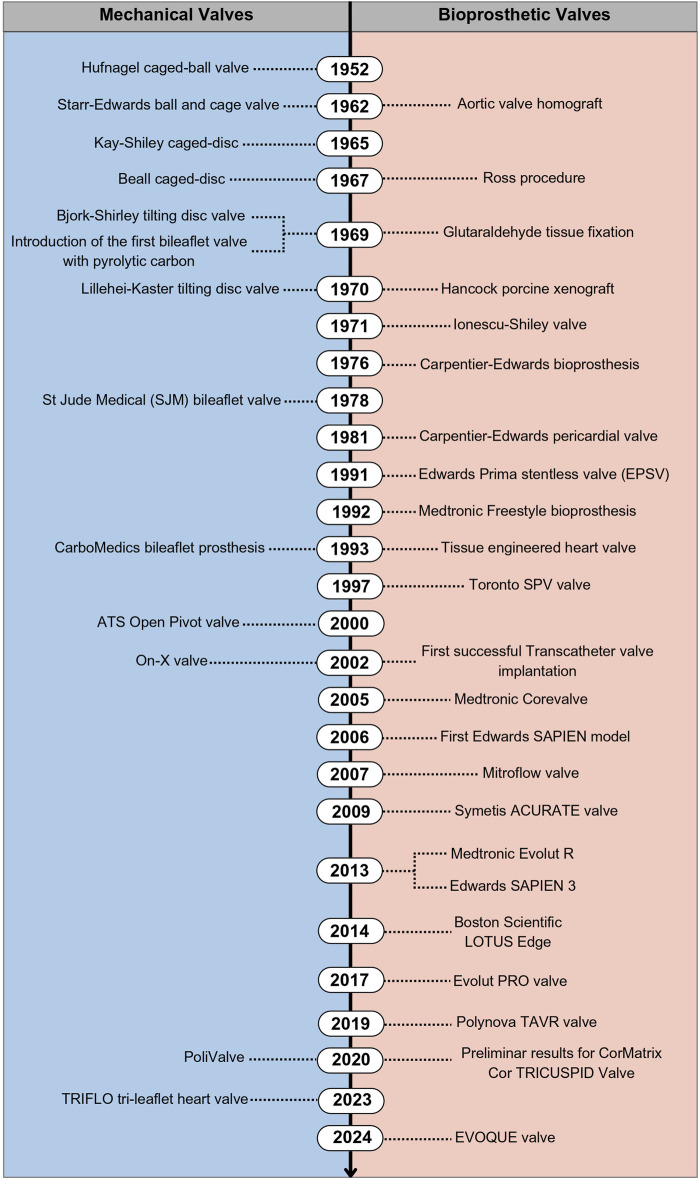
Chronology of the advances in mechanical and bioprosthetic valves.

In 1923, the first heart valve surgery occurred to repair a cardiac valve in a 12 year-old child who suffered from mitral stenosis (Cutler and Levine, 1923) by dilating the orifice of the valve. According to Lodhia and Evans (Lodhia and Evans, 2018), this procedure was effective only for this child (five patients could not survive). The first replacement of a diseased valve occurred in 1952. Since then, many artificial and biological valves have been tested. Figure 3 shows a chronology of the types of valves implanted, based on (Fioretta et al., 2021; Dasi et al., 2009; Marco et al., 2017).

In 1952, the first prosthetic heart valve replacement used a Hufnagel caged-ball valve. Rather than removing the diseased aortic valve, the clinical team inserted it in the patient’s descending aorta. This innovation provided a step toward introducing a heart-lung bypass machine to perform an atrial septal repair in 1953. In the 1960s, bioprosthetic valves became an alternative to mechanical valves (Head et al., 2017). In 1962, cadaveric aortic valves were used after cryogenics or with antibiotics; however, since they required a cadaveric valve supply, this alternative was considered far from ideal. Still in 1962, a new mechanical valve called Starr-Edwards ball-and-cage valve replaced the Hufnagel caged-ball since the former introduced a ball inside a metal cage to prevent backflow into the valve orifice. Then, the Starr-Edwards valve received modifications to improve its performance, e.g., the type of caged disc, such as the Kay-Shiley (1965) and Beall prostheses (1967). Despite the change from a ball to a disc, the medical team suspended this new design due to its inferior hemodynamic characteristics. Thus, some surgeons still opt for the Starr-Edwards valve.

In 1967, a new technique called the Ross procedure was introduced, which replaced diseased aortic valves with patients’ own pulmonary valve (autograft), substituting the latter with a prosthesis. The year of 1969 was marked by two importants breakthroughs in both mechanical and bioprosthetic valves. For tissue valves, in order to improve the stability and decrease biological tissue biodegradation glutaraldehyde tissue fixation technique was introduced, this advance fostered the use of several tissues (such as porcine valves) and pericardium tissues. For mechanical valves, the Björk-Shiley tilting disc valve introduced a new function mechanism by having discs blockading their orifices when closed and remaining inclined when open. Also in 1969, the first bileaflet mechanical heart valve (with pyrolytic carbon) was produced (Bokros et al., 1969), a biomaterial used to this day.

In 1970, the Hancock Porcine Xenograft (Medtronic, Irvine, CA, USA) and Lillehei-Kaster tilting-disc valve became commercially available. In 1971, a new bioprosthetic valve (Ionescu-Shiley) was produced and implanted, only reaching the market in 1976. This bioprosthesis was developed from bovine pericardium tissue with a Delirin flexible stent for its leaflet structure, showing better symmetrical hemodynamics than porcine valves. However, it considerably deteriorated over time and its production ceased in 1980 (Marco et al., 2017). 1976 also introduced a popular bioprosthetic valve on the market, the Carpentier-Edwards Bioprosthesis (Edwards Lifesciences, Irvine, CA, USA), which uses porcine tissue with a flexible support frame.

The St Jude Medical Inc. (Minneapolis, MN, USA) was produced with pyrolytic carbon and was the first of this kind to arrive on the market in 1978. The following 14 years were marked by bioprosthetic valve improvements. In 1981 the clinical use of calf pericardial tissue with the Carpentier-Edwards calf pericardial valve was approved, being commercialized in the US in 1991, followed by the Edwards Prima stentless valve (EPSV) (clinical trials in 1991) and the Medtronic Freestyle (approved in 1992 for human trials). Only in 1993, after bileaflet mechanical heart valves, several improvements occurred, such as the CarboMedics bileaflet prosthesis which differs from that used in the St Jude Medical regarding shape and opening angle of its leaflet.

Langer and Vacanti (1993) obtained a tissue-engineered heart valve (TEHV) by autologous cells seeding in a biodegradable polymer scaffold. They formed their tissue in an *in vitro* bioreactor and grew and remodeled it *in vivo* after implantation. Until 1997, leaflet tissues were believed to be responsible for the mechanical stability of valves. Thus, bioprostheses were fixed in metallic stent supports. On the other hand, with the introduction of stentless bioprostheses, researchers found that mechanical stability may be distributed across the aortic root, dispensing with the need for a stent. The FDA approved the Toronto SPV valve (St Jude Medical) — the first stentless heart valve—in 1997.

In 2000, the ATS Open Pivot valve emerged differing from previous valves due to its design pivot. The most recent mechanical valve design introduced in the US (FDA approved in 2002) was the On-X valve—marketed by the Medical Carbon Research Institute (Austin, TX, USA) —, a new generation of bileaflet valves (Chaudhary et al., 2017). It remains the most popular mechanical valve, differing from others by its length-to-diameter similarity with native heart valves.

In 2007, the FDA approved a new pericardial tissue-based bioprosthetic valve, the Mitroflow valve by the Sorin Group Canada Inc. (Burnaby, BC, Canada). In 2002, Cribier et al. (2002) reported a new surgical advance, a less invasive procedure using a metallic scaffold to percutaneously implant heart valves into patients, known as transcatheter aortic valve implantation (TAVI) or replacement (TAVR). This technology has greatly advanced medicine, configuring a novel option to treat patients with high surgical risk or with multiple comorbidities (Fioretta et al., 2021). Transcatheter bioprostheses have a metallic frame which, once deployed into damaged native valves, attach and fix prostheses to vascular walls. Since the first transcatheter implantation in 2002, other devices emerged, such as the Medtronic Corevalve (2005), the first Edwards SAPIEN model (2006), and the Symetis ACURATE valve (2009).

To improve the design of catheter delivery systems and minimize leaflet calcification and deterioration, modifications and treatments were tested, such as fixation in glutaraldehyde and other anticalcification processes, culminating in new prostheses, such as the Medtronic Evolut R (2013), the Edwards SAPIEN 3 (2013), Boston Scientific LOTUS Edge (2014) and, most recently, Evolut PRO valve (2017) (Marco et al., 2017).

In 2020, Stasiak et al. (2020) introduced a promissory prosthetic heart valve developed and tested under ISO standards, the PoliValve. The authors manufactured their prototypes with commercial block copolymers, producing safe, biocompatible, and durable structures capable of resisting over 1.2 billion cycles. In 2023, Carrel et al. (2023) introduced a new tri-leaflet mechanical valve called TRIFLO. This prosthesis consists in a structure of titanium with three rigid polymer (PEEK) cusps. The pre-clinical studies performed in sheep demonstrated no significant difference between TRIFLO valve and control (One-X valve), the results were favorable but the authors emphasize that only implantation and long-term observation in humans can confirm these results. The Edwards EVOQUE valve is the most recent tricuspid valve replacement system approved by FDA (approval date in 2024). It is composed of an artificial valve made with a cow tissue attached in a self-expanding metal (nickel-titanium) frame and a delivery catheter, it is intended to replace the tricuspid valve without open-heart surgery.

Over the years new technologies and applications for cardiac valve prosthesis occurred, some studies demonstrated promising results using biological and polymeric materials. In 2019, Rotman et al. (2019) manufactured a second-generation valve (Polynova TAVR valve) for aortic replacement using polymer xSIBS (cross-linked SIBS (poly-(styrene-blockisobutylene-block-styrene)) and the achieved results showed low thrombogenic potential, calcification resistance and efficient hemodynamics (Singh et al., 2023). In 2020, Gerdisch et al. (2020) published early results from FDA clinical feasibility trial of a bioprosthetic tricuspid valve, the CorMatrix Cor TRICUSPID Valve. It was found an excellent valve function with remodeling native tissue and no calcification in short-term analyses. Movileanu et al. (2021), in 2021, produced decellularized ovine pulmonary valves by perfusion, cell-seeded with differentiated autologous adipose-derived stem cells and pursued orthotopic implantation in an ovine animal model. After 6 months and valve explantation, the authors relate that the pulmonary valve implanted was intact, with no evidence of rejection.

Finally, the development of mechanical heart valves with better hemodynamics and low thrombogenicity is still necessary. This valve’s great advantage is its durability when compared to bioprosthetic valves, on the other hand, bioprosthesis do not need long-term anticoagulation but are more susceptible to structural degeneration (Singh et al., 2023). New materials have been studied for valve prosthesis applications that allow large-scale development at low cost, such as polymers (Rotman et al., 2019; Singh et al., 2023). In this context, there is still no cardiac valve prosthesis on the market that meets all the functionality requirements, one example is the need for pediatric valve prostheses that offer better adaptation to the patient’s growth (Movileanu et al., 2021).

### 4.1 Technological milestones in the evolution of cardiac valve prostheses

In general, the technological evolution in heart valve replacement is marked by disruptive advancements that have transformed cardiac care. In 1952, the introduction of the Hufnagel valve, the first mechanical prosthesis, provided innovative solutions for previously untreatable conditions. The 1960s drove biotechnology forward with the use of biological tissues, culminating in the Ross procedure in 1967, which pioneered the concept of autografts. The breakthroughs of 1969, such as glutaraldehyde fixation and the Björk-Shiley valve, elevated hemodynamic performance, setting the stage for more refined solutions.

The following decades witnessed the rise of stentless valves like the Toronto SPV, which revolutionized mechanical stability by eliminating metallic supports. The 2000s were pivotal with the advent of TAVI, introducing minimally invasive replacements for high-risk patients. Devices like the SAPIEN and Evolut PRO enhanced safety and efficacy, while advanced biomaterials extended prosthetic durability.

Recently, solutions like the PoliValve (2020) and TRIFLO (2023) represent the pinnacle of valve engineering, promising to merge durability, biocompatibility, and physiological adaptation. However, challenges such as pediatric prostheses and calcification resistance still drive innovation. The field continues to explore novel materials and customizable designs, aiming to meet all clinical needs. In the next sections, we will discuss the biomechanics and hemodynamics of prosthetic valves, types of valvular prostheses, perspectives for overcoming remaining limitations, and future trends in the field.

## 5 Biomechanics and hemodynamics of prosthetic valves

It is well known that prosthetic valves play a crucial role in treating heart diseases by replacing defective natural valves to restore proper cardiovascular function. However, the interaction of these prostheses with the cardiovascular system must be thoroughly analyzed, as several factors influence their performance, including valve design, blood flow dynamics, structural integrity under cyclic stress, and key metrics such as effective orifice area, pressure gradient, and regurgitation rate.

The design of prosthetic valves is vital for ensuring effective biomechanical and hemodynamic functionality. Danilov et al. (2023) explored the application of machine learning and generative design, demonstrating how geometric customization optimizes pressure gradients, blood flow, and reduces structural stresses. Ghanbari et al. (2022) emphasize the importance of fluid-structure interaction (FSI) simulations to assess and adjust leaflet stresses, preventing mechanical failures. Both studies underscore that meticulous design, combined with advances like 3D printing, enhances durability and safety by tailoring prostheses to specific physiological conditions. These innovations highlight the importance of robust design in reducing clinical complications such as thrombosis and regurgitation, ultimately improving patient outcomes.

Analyzing flow dynamics and resistance is fundamental, as it directly impacts the hemodynamic performance and longevity of prosthetic valves. Blood flow behavior around valves, including vortex formation and separation zones, is associated with complications such as thrombosis and regurgitation. Yoganathan and Sotiropoulos (Yoganathan and Sotiropoulos, 2004) demonstrated that Computational Fluid Dynamics (CFD) is essential for examining three-dimensional flow patterns and identifying regions of critical stress, aiding in valve design optimization. Laubscher et al. (2022) added to this perspective by investigating how different valve geometries influence flow resistance and vortex formation, highlighting the need for geometric adjustments to enhance hemodynamic compatibility.

Furthermore, Collia et al. (2021) emphasized the importance of considering right ventricular dynamics, showing that changes in flow behavior can impact valve performance. Snyder and Jana, (2023), in turn, focused on the biomechanics of valves, exploring how material and structural choices affect mechanical resistance and durability under cyclic stress. Collectively, these studies underscore that the integration of advanced techniques, such as CFD and fluid-structure analyses, is indispensable for developing safer and more efficient valves. By thoroughly exploring the interaction between flow and resistance, it is possible to design valves that optimize hemodynamic performance and minimize clinical complications.

Structural integrity under cyclic stress is a critical aspect in the development of prosthetic heart valves, as these devices are subjected to repetitive forces during the cardiac cycle, directly impacting their durability and safety. Dayawansa et al. (2022) underscored how mechanical stress contributes to calcification in aortic valves, degrading structural integrity over time. Sadeghinia et al. (2023) utilized computational models to identify stress concentration areas that may lead to structural failure. Pedersen et al. (2023) applied experimental and fluid-structure interaction techniques to assess the mechanical response of biological valves, providing valuable data for design optimization. In general, these studies underscore the importance of integrating experimental and computational analyses to enhance valve design, ensuring greater resistance to cyclic stress and improved hemodynamic performance, which is essential for prosthesis longevity.

The analysis of performance metrics for prosthetic heart valves is crucial to assess their functionality and prevent complications such as stenosis, regurgitation, and prosthesis-patient mismatch. Brandt and Pibarot (2021) emphasize the role of echocardiography in measuring the effective orifice area (EOA), which is essential for evaluating transvalvular flow efficiency and detecting prosthetic stenosis. Franz et al. (2021) highlight the importance of indexing EOA to body surface area, suggesting that this approach can enhance the assessment of hemodynamic compatibility. Toma et al. (2022) demonstrate how computational methods aid in analyzing pressure gradients and regurgitation rates, providing a more detailed understanding of valvular flow dynamics. These studies underscore that integrating imaging techniques, clinical analyses, and computational simulations is critical for the development of more effective and safer valves, ensuring improved hemodynamic performance and enhanced durability.

## 6 Types of valvular prosthesis

Studies have considered many factors in choosing valvular prostheses, e.g., patients’ age, coagulation issues, intolerance to medications, survival expectancy, and others (Cheung et al., 2015; Lodhia and Evans, 2018). Choices generally depend on medical teams and patients discussing the risks and benefits of each option (Baumgartner et al., 2017). Thus, patients must remain safe and be aware of the properties, advantages, disadvantages, and life expectancy each valve may offer them.

### 6.1 Mechanical valves

Durability constitutes the greatest advantage of mechanical valves, minimizing patients’ reoperation since they can last up to 30 years (Fiedler and Tolis, 2018). The material commonly used in mechanical valves is pyrolytic carbon. Originally serving to encapsulate nuclear fuel rods, carbon was adapted for the fabrication of mechanical valves discs, leaflets, and housings (Cheung et al., 2015; Fiedler and Tolis, 2018; Bokros et al., 1969). Studies have deemed pyrolytic carbon as a good biomaterial to manufacture mechanical valves due to its hemocompatibility, durability, and good mechanical properties (Wium et al., 2020). However, it may contain a small amount of silicon to stiffen the valve, and thus cause platelet aggregation with thrombus formation (Chaudhary et al., 2017). Thus, consensus suggests the need of investigating other biomaterial types to manufacture mechanical valves which would improve this limitation (Wium et al., 2020).

Due to the thrombogenicity mechanical valves induce, patients may depend on lifelong oral anticoagulation therapy, which may cause spontaneous bleeding episodes (Cheung et al., 2015; Head et al., 2017). Vahanian et al. (2021) Guidelines for the management of valvular heart disease recommend that mechanical prostheses should be considered in as an alternative for patients already on anticoagulation medication for several diseases, those under high risk of thromboembolism, those showing a risk of accelerated structural valve deterioration, those with a reasonable life expectancy and high reoperation risk, those aged over 60 years (in the case of aortic positioning), and those aged over 65 years (in cases of mitral prostheses).

According to Sotiri et al. (2019), research assessing the interaction between synthetic biomaterials (e.g., mechanical valves) and blood has declined since the progress to understand hemocompatibility has been slow. In this sense, interventions with anticoagulation therapies became common, configuring the sole treatment to heal artificial valve limitations and saving and improving patients’ quality of life. However, they still have well-recognized significant limitations. Thus, researchers on biomaterials should prioritize exploring the intractability between blood compatibility and synthetic materials.

### 6.2 Bioprosthetic valves

Bioprosthetic valves (also known as biological or tissue valves) consist of porcine valves or bovine pericardial tissue, requiring treatment by a variety of methodologies to neutralize them and exclude any immunogenicity to render valve xenotransplants viable (Cheung et al., 2015; Fiedler and Tolis, 2018). Their use stems from their mimicry of the tri-leaflet morphology, (as Figure 1 illustrates), leaflet natural/closing functions, and hemodynamic profiles (Cheung et al., 2015; Fiedler and Tolis, 2018). Despite similarity between their structural morphologies, the elastic modulus of porcine aortic valves is lower than native valves, for example, which may reach 7.8 MPa—a difference of about 52% (Li et al., 2019). A considerable advantage they have is their lower thrombogenicity, avoiding patients’ lifelong anticoagulant use (Fiedler and Tolis, 2018; Head et al., 2017). Additionally, bioprosthetic valves are the only option that a minimally invasive transcatheterism can implant. Since it reduces invasiveness, this technique has aroused the interest of medical teams to more widely use them for patients of all ages and expand mechanical valves (Cheung et al., 2015; Fiedler and Tolis, 2018; Head et al., 2017).

The major risk of bioprosthetic valves is reoperation since their structure may deteriorate after 10–20 years in older adults. This reduction in biomaterial durability remains unclear; however, it is supposed that it may stem from the calcium in their extracellular fluid reacting with the phosphorus associated with the membrane, calcifying cells. Younger patients and children generally show accelerated calcification due to their higher metabolism and stronger immunologic response (Cheung et al., 2015; Head et al., 2017; Schoen and Levy, 2005). Also, studies have suggested that glutaraldehyde, the most common biological valve preservative, may also accelerate calcification since it has been hypothesized that it modifies phosphorus-rich calcified structures in valve tissues, causing mineralization (Schoen and Levy, 2005). Thus, Vahanian et al.’s guidelines for the management of valvular heart disease recommended the aortic position implantation of bioprosthetic valves in patients over 65 years and mitral position in patients over 70 years (Vahanian et al., 2021). According to Hoffmann et al. (Hoffmann et al., 2008), the degeneration of bioprosthetic valves in the mitral position occurs more often than in the aortic position due to their greater systolic vs. diastolic hemodynamic demand.

Fiedler and Tolis (2018) reported that, due to their structural deterioration and subsequent calcification, commercial valve companies have explored anticalcification treatments, including exploring valves in detergents to catalyze the lysis of the bonds between calcium and phospholipids or other compounds that aim to alter the structure of aldehyde groups. Moreover, second-generation pericardial bioprosthetic valves have also been fabricated to minimize calcification but results have failed to show improvements.

## 7 Perspectives for reminiscent limitations of valvular prosthesis

Both mechanical and biological valves show risks for patients, thus requiring extensive research on solutions for such problems. The following subtopics describe the existing perspectives to improve the limitations of valvular prosthesis replacement.

### 7.1 Biomaterials for mechanical valves

Mechanical valves employ selected synthetic materials in their production, such as metal alloys, graphite, and polymers due to their mechanical strength and durability. Moreover, the resistance of synthetic materials to reactions with foreign bodies must also be considered. Metal alloys, such as titanium or stainless steel, show higher thrombogenicity rates. Thus, pyrolytic carbon, a graphene-based material, has been used due to its appropriate mechanical characteristics and decreased thrombogenicity (Atcha and Liu, 2019).

Despite the advantage pyrolytic carbon shows compared to other synthetic materials, it may contain subproducts that cause thrombosis. Thus, patients would require lifelong anticoagulation therapy, which may cause excessive bleeding and impair blood clotting, hence the need for a new biomaterial to overcome this limitation. However, research in this area remains limited. Scientists find non-degradable polymers, for example, as an alternative biomaterial to produce heart valves due to their durability and physiological hemodynamic profile. Nevertheless, despite the ongoing progress in polymeric valve development, results remain clinically unacceptable (Atcha and Liu, 2019; Fioretta et al., 2018). Moreover, research must seek a biomaterial with excellent resistance to mechanical and structural wear (Vellayappan et al., 2015).

### 7.2 Tissue-engineering heart valve

Due to the limitations of valvular prostheses, tissue-engineered heart valves (TEHVs) configure an innovative alternative as they use a scaffold in place of conventional valves since they are three-dimensional platforms that proliferate, grow, and differentiate cells. Scaffolds should have a suitable morphology mimicking that of anatomical valves and satisfactory mechanical properties. However, reproducing this complex structure is a challenge since it requires cells to behave ideally given their interactions with their surroundings, such as physical (fibronectin, collagen, glycosaminoglycans, etc.) and soluble signals (growth factors, cytokines, small molecules, ions, etc.), cell-cell interactions, and mechanical, pH-related, and oxidative stress (Boroumand et al., 2018). Moreover, heart valve scaffolds must open and close correctly, be biocompatible, non-immunogenic, non-thrombogenic, easily replaceable, and non-obstructive (Cheung et al., 2015; Jana et al., 2014).

Several types of materials are currently investigated as potential scaffolds for heart valves, such as fabricated materials, allogenic and xenogeneic heart valves, and others, such as polymers. The techniques to produce scaffolds generally comprise decellularization, molding, electrospinning, and 3D bioprinting (Cheung et al., 2015; Fioretta et al., 2018).

#### 7.2.1 Allogenic and xenogeneic biomaterials

Non-fabricated biomaterials, such as those in allogeneic and xenogeneic heart valves, show adequate biological properties and excellent anatomic structure (Boroumand et al., 2018). Allogeneic heart valves have excellent hemodynamic performance and good functionality, especially after cell implantation. However, xenogeneic heart valves have received great attention due to donor scarcity. Porcine or bovine pericardial tissue valves offer attractive sources of unlimited materials, providing a viable and functional alternative. However, these biomaterials show disadvantages, such as low cellular infiltration and possible calcification, the main reason for the changes to non-fabricated prosthesis with a lifespan between 10 and 20 years (Fioretta et al., 2018).

In general, to avoid any rejection, allogeneic and xenogeneic tissues must undergo decellularization (Naso and Gandaglia, 2018) to remove viable cells and immunogenicity and retain the viability of the extracellular matrix. This may come about by combining physical, chemical, and enzymatic methods. Unlike extracellular matrix preservation, decellularization can degrade components and affect their structural integrity. Furthermore, xenogeneic tissue decellularization may show residual cells, DNA, and the α-Gal epitope, which induces an inflammatory response or immune-mediated rejection (Atcha and Liu, 2019; Boroumand et al., 2018).

After decellularization, biological cardiac valves undergo chemical stabilization (glutaraldehyde fixation), providing excellent hemodynamics and low thrombogenicity and improving handling and especially long-term stability (Vincentelli et al., 1998). However, glutaraldehyde can cause low cellular infiltration and calcification. Its residuals may hinder *in vivo* recellularization due to their cytotoxic effects. Moreover, glutaraldehyde preserves residual endothelial cell membranes or fragments from donors, conserving the remaining phosphorus. Thus, in the absence of mineralization inhibitors, calcium phosphate crystals nucleate and grow, causing calcification. In this sense, alternatives for these limitations include complete glutaraldehyde removal and *in vitro* endothelization (Atcha and Liu, 2019; Lee et al., 2017).

The recellularization of bioprosthetic valves is the better option for the longevity and efficiency of decellularized valves since it can decrease platelet adhesion and retard calcification but this alternative remains a major challenge (Atcha and Liu, 2019; Zhou et al., 2019).

The absence of endothelial cells in decellularized valves may expose their surface fibers, which could activate blood platelets via adhesion and aggregation. Given this information, Zhou et al. (2019) evaluated decellularized porcine aortic valve biofunctionalization by covalent modification to accelerate endothelialization. The authors developed a hybrid system with polycaprolactone nanoparticles, encapsulating a vascular endothelial growth factor modified by maleimide-poly (ethylene glycol)-b-poly (ε-caprolactone). Their results showed accelerated endothelization with no significant blood platelet adhesion after nanoparticle modification. Dai et al. (2019) covered a decellularized porcine aortic valve leaflet with a modified porous matrix of metalloproteinase degradable poly (ethylene glycol) hydrogel modified containing stromal cell-derived factor-1α. This strategy assisted bone marrow mesenchymal stem cell adhesion, viability, and proliferation in decellularized tissues and promoted their differentiation into valve interstitial-like cells. Their thrombogenicity assay showed that tissues with hydrogel modification presented a significantly decreased platelet adhesion without activation. Furthermore, they observed no visible calcification in formulations with or without modified hydrogel. Although these studies showed promising results, a positive outcome for the long-term success of any bioprosthetic heart valves after testing in an *in vivo* hemodynamic environment, for example, remains published.

#### 7.2.2 Xenografts from genetically modified pigs

The galactose-α1,3-galactose epitope (α-gal) is a carbohydrate present in proteins and lipids of non-primate mammals (Perusko et al., 2024), synthesized by the glycosylation enzyme α-1,3-galactosyltransferase (α1,3 GT), encoded by the GGTA1 gene (Galili, 2005; Zhang et al., 2018). This epitope has been reported as the major xenoantigen responsible for immune rejection and hyperacute rejection of xenografts (Perusko et al., 2024; Sandrin and McKenzie, 1994; Carvalho-Oliveira et al., 2021; Galili, 2001), explaining its clinical relevance.

The human body naturally produces anti-Gal antibodies and a promising alternative to bypass xenotransplantation rejection was created by α-1,3-galactosyltransferase gene-knockout (GTKO) in pigs (Sandrin and McKenzie, 1994; Montgomery et al., 2022). This genetic alteration was first published in 2003 (Phelps et al., 2003) and recently approved for both food and as a potential source for biomedical use by the Food and Drug Administration (FDA). Montgomery et al. (Wium et al., 2020) demonstrated two cases of pig-to-human kidney xenotransplantation. His study used GTKO pigs with subcapsular autologous thymic tissue (called a “thymokidney”) to promote immune tolerance and reduce risk of late rejection, after transplantation the organs were monitored for 54 h, then explanted. The “thymokidneys” were able to produce urine in the first minutes, the authors suggested that the elimination of α-gal residues alone prevented both recipients hyperacute rejection (Montgomery et al., 2022).

In attempt to enhance the suitability of GTKO pigs, additional genetic modifications were made like cytidine monophosphate-N-acetylneuraminic acid hydroxylase (CMAH), beta-1,4-N-acetyl-galactosaminyltransferase 2 (β4GalNT2), and growth hormone receptor (GHR) genes knockouts, and insertion of human CD46, CD55, CD47, thrombomodulin (THBD), protein C receptor (PROCR) and heme oxygenase 1 (HMOX1) transgenes (Peterson et al., 2024; Judd et al., 2024; Eisenson et al., 2024). Organs from pigs with these 10 genetic edits (10 GE) were already used in pig-to-nonhuman primate (NHP) and pig-to-human xenotransplantation (Peterson et al., 2024; Judd et al., 2024).

Heart valve xenotransplant using genetic modified pigs as donors are an alternative to reduce implant degradation and rejection (Zhang et al., 2018). Two important antigens from porcine heart valve described are α-gal and N-glycolylneuraminic acid (NeuGc), the last one is synthesized by cytidine monophospho-N-acetylneuraminic acid hydroxylase (CMAH) (Zhang et al., 2018; Hutton et al., 2024).

Lee et al. (Danilov et al., 2023) studied human antibody recognition for xenoantigens from porcine heart valve with GTKO/CD46 and GTKO/CD46/NeuGc genetic modifications and compared with porcine heart valves from wild-type (WT), glutaraldehyde-fixed bioprosthetic heart valves (GBHV) and human heart valve. They found that human IgM and IgG antibodies binding decreased for both heart valves from genetic modified pigs when comparable to WT, however, the GTKO/CD46/NeuGc pig valves binding was comparable to human valves (Lee et al., 2016). Zang et al. (Bokros et al., 1969) analyzed porcine pericardium from GGTA1, CMAH, and β4GalNT2 triple gene-knockout (TKO) pigs and concluded that IgG/IgM antibody binding was reduced as in human valves.

The application of genetic modified pigs in valve xenotransplantation was already shown, however further studies are needed to determine the ideal genetic modification to avoid rejection and the structural valve deterioration (SVD) caused by calcification in bioprosthetic heart valves.

#### 7.2.3 Fabricated heart valves

The advantages of fabricated heart valves include their rapid manufacturing, low cost, predictable degradation rate, and hemodynamic resistance (Oveissi et al., 2020). Scientists always seek materials to replicate the complexity of native valves with appropriate cell seeding, differentiation, and remodeling (Cheung et al., 2015). Polymers or their composites offer a promising alternative for a valve heart design due to their ability to mimic native valves, reducing thrombosis and showing higher durability. Thus, they may overcome mechanical and bioprosthetic valves (Li et al., 2019). However, only mechanical and bioprosthetic valves are used in clinical application today (Oveissi et al., 2020).

According to Boroumand et al. (2018), both synthetic [polyglycolide, polylactic acid, poly (lactic-*co*-glycolic acid), poly-L-lactic acid, polyethylene glycol, polycaprolactone, polyanhydrides, and polycarbonates] and natural polymers (collagen, hyaluronic acid, and chitosan) may serve as heart valve scaffolds. Synthetic polymers show reproducibility and easy architecture control and can improve their mechanical properties by combining with other polymers. However, their degradation may release toxic subproducts. Natural polymers have greater biomimetic and natural cellular adhesion sites but have reproducibility difficulties and inadequate mechanical properties.

Another promising alternative is reinforcing materials with nanoscale fillers (nanocomposites), which are commonly incorporated into polymeric materials to improve their mechanical and chemical properties. However, experimental and clinical studies remain inconsistent since they are unable to reproduce several nanocomposites (Vellayappan et al., 2015). Therefore, the success of fabricated materials in TEHV will depend on their application results, when compared with conventional valvular prostheses (Fioretta et al., 2018).

Producing fabricated heart valves includes molding, electrospinning, and 3D bioprinting (additive manufacturing). Oveissi et al. (2020) clearly describe these techniques to produce heart valves. In general, molds with the desired geometry receive a polymeric solution or melt that solidifies before their release. Electrospinning produces nanofibers by electrostatic repulsion, ejecting them by a wire producer (e.g., syringe) loaded with a polymeric solution or melt that has been subjected to a high voltage electric field. Three-dimensional bioprinting may create complex cellular structures with defined details. There are three methods of 3D biofabrication: laser-induced forward transfer, inkjet, and extrusion printing. Among these techniques, extrusion printing or bioplotting is the most flexible for design heart valves. This method includes dispensing bioinks through a nozzle on a platform and controlling its deposit into *x*, *y*, and *z* coordinates. Table 3 summarizes the advantages and disadvantages of each technique.

**TABLE 3:** Comparison of fabrication techniques (molding, electrospinning, and 3D bioprinting), outlining their strengths and limitations for valve engineering.

Techniques to produce fabricated heart valves	Advantages	Disadvantages
Molding	- Use of different polymers in the same mold to make multi-material structures - different types of cells and materials can be applied - high viability for incorporated cells	- Complications and discrepancies in manufacturing due to the multistep process that needs to opening and closing the mold several times - isotropic mechanical properties in each phase
Electrospinning	- Highly versatile and efficient technique - easily scaled up - can be controlled to create mechanical anisotropy - allow desirable macroscopic shapes and sizes, mechanical properties, heterogeneity, and microstructures	- Cells are unable to be directly used in electrospinning due to the presence of organic solvents and high voltage
3D bioprinting	- Versatility and capability to supply scaffolds containing cells with controlled structure over spatial distributions Bioplotting - The ability to deposit more than one material in each layer - versatility, stackability, and the possibility of loading a high density of cells into the bioink - the ability to deposit multimaterials at high throughputs	- Difficulty to adapt bioinks to obtain well-defined constructions with suitable conditions for cells Bioplotting - Resolution is lower than other methods

Although preclinical studies were conducted only with heart cast or electrospun valves, 3D bioprinting offers many opportunities since it may solve the discrepancy between fabricated tissue and native ones. The technique can build heterogeneous structures with more suitable anisotropy. However, bioinks require optimization to be highly functional in cells. Additionally, bioprinted cardiac valves are unable to open and close without a heart cardiac cycle. Thus, bioprinting cardiac valves still await human tests (Oveissi et al., 2020; Zhang et al., 2017).

#### 7.2.4 Functionalization of valvular bioprostheses

The current panorama to solve prosthesis limitations focuses on cell adhesion, growth, and differentiation capacity of scaffolds. For that, researchers have been studying modifications to scaffolds by adding bioactive molecules via covalent or non-covalent bonds, i. e., “functionalization” (Fioretta et al., 2021; Taylor, 2007). Studies have evaluated some biomolecules for scaffold functionalization, such as transforming growth factor-β (TGF-**β**), vascular endothelial growth factor (VEGF), stromal cell-derived factor-1α, and the RGD peptide (Filova et al., 2021). Bensimon-Brito et al. (2020) used a suitable animal model (adult zebrafish) to identify modulators for implanted valves and found that TGF-**β** plays an important role in AV regeneration since it regulates endothelial cell proliferation and endothelial to mesenchymal transitions, contributing to VIC differentiation.

Since VEGF improves cell proliferation and adhesion, it has been used to the endothelization of decellularized scaffolds (Lei et al., 2019; Neufeld et al., 1999). In this perspective, Marinval et al. (2018) showed that a Fucoidan/VEGF-based polyelectrolyte multilayer film which continuously released VEGF applied to the surface of decellularized pulmonary heart valves decreased thrombogenicity and coagulation and promoted remodeling by increasing cell adhesion and viability. Lei et al. (2019) compared endothelization in porcine pericardium fixed in glutaraldehyde (GLUT) as a control group with hybrid pericardium coated with hyaluronic acid hydrogel (GLUT/HA) and hybrid pericardium tissue coated with VEGF-loaded hyaluronic acid hydrogel (GLUT/HA/VEGF). The results showed improved cell adhesion and proliferation and decreased platelet adhesion and calcification in GLUT/HA/VEGF. Thus, they considered this type of functionalization as a promising alternative for applying these bioprosthetic valves in clinical applications.

Another molecule extensively used to functionalize biological tissues is the RGD peptide, which consists of arginine, glycine, and aspartic acid in sequence. Studies showed that the RGD peptide can improve cell adhesion and endothelialization and that its association with VEGF can refine those responses (Porter et al., 2011). Shi et al. (2009) showed myofibroblast adhesion in valve scaffolds functionalized with RGD, describing it as an interesting technique for *in vitro* tissue engineering of heart valves. Zhou et al. (2016) used endothelial progenitor cells to repopulate *in vitro* decellularized valves containing RGD and VEGF and noted that functionalization improved endothelial progenitor cell adhesion and proliferation and induced the formation of a functional layer of endothelial cells.

Functionalization offers a new and promising TEHV strategy. However, further studies must evaluate new molecules and techniques to functionalize heart valve scaffolds to reach initial *in vivo* assays in large animals.

### 7.3 Transcatheter heart valves (THV)

For many decades, conventional open-heart surgery has offered an option to replace natural valves, but this approach may be fatal for patients with comorbidities (hypertension, respiratory insufficiency, peripheral arterial stenosis, chronic renal failure, etc.) (Fioretta et al., 2018). Thus, since 2002, a new alternative has been a trend; a less invasive procedure that neither demands general anesthesia, causes cardiac arrest, extracorporeal circulation nor requires other forms of circulatory support.

THV involves replacing diseased or damaged heart valves with a catheter containing a new valve via a minimally invasive procedure, such as a small incision in patients’ groin or shoulder. The aortic valve implantation usually occurs by transcatheter aortic valve implantation (TAVI); the success of this new approach generated interest in applying this technology to mitral and tricuspid valves (Tabata et al., 2019). THV require crimping the valve replacement, meaning, thus, that only bioprostheses are suitable for this procedure, generating an important advantage for patients that receive it since it allows the re-operation of bioprosthetic valves after their degeneration with less invasiveness and faster recovery time (Head et al., 2017; Fioretta et al., 2018). However, crimping may be a limitation for THV since it could generate irreversible mechanical damage during it and deployment (Rotman et al., 2020). Furthermore, due to the difficulty of controlling the properties of decellularized matrices, mechanical valves have offered a promising option since they can control length scale and show extraordinary reproducibility (Stassen et al., 2017). Regarding the mechanical valves that are suitable for THV, medical teams can approve younger patients for this procedure (Head et al., 2017). Recent studies have assessed the perspective of using polymeric TAVI to remedy the limitations due to bioprosthetic and mechanical valves. Additionally, polymeric materials may show better resistance to crimping, allowing its mass manufacture due to their high reproducibility and lower overall manufacturing costs (Rotman et al., 2020; Ghosh et al., 2018).

## 8 Future perspectives

The future prospects for the development of heart valves are highly promising, driven by emerging innovations aimed at enhancing available treatment options. An essential component of these advancements is the role of artificial intelligence (AI) in diagnosing VHDs, a field that is rapidly gaining traction. With the integration of AI into medical equipment, diagnostic accuracy has reached new levels. AI-powered devices, such as electronic stethoscopes and intelligence-augmented ECG monitors, leverage clinical data and advanced algorithms to identify, extract, and analyze critical features, including low-frequency sound waves and cardiomechanical signals, with a precision that surpasses traditional, non-AI-enabled machines (Sengupta et al., 2024). These innovations not only enhance early detection but also provide deeper insights into disease progression, paving the way for more targeted and effective treatment strategies.

Building on these advancements in diagnostic technologies, the development of next-generation heart valve prostheses is taking center stage. A noteworthy example is the work of Brazilian researchers from the University of São Paulo, who are refining the Wheatley Aortic Valve, an advanced polymeric prosthesis designed for patients with aortic stenosis. This valve offers a significant advantage: the potential to eliminate the need for postoperative anticoagulants, simplifying clinical management and reducing associated risks. The development process involves cutting-edge mathematical modeling and computational simulations to optimize the valve’s performance before moving on to clinical trials (Usp, 2023).

While the development of the Wheatley Aortic Valve represents a breakthrough in polymer-based prostheses, researchers are also investigating hybrid heart valves as an alternative pathway to address the complex demands of valve replacement. These hybrid designs aim to leverage the best features of mechanical and biological valves, offering a compelling solution to bridge the gap between durability and biocompatibility. Nazir et al. (2021) investigated the efficacy of hybrid scaffolds in valve engineering, highlighting that material combinations can yield superior mechanical properties and controlled degradation rates, critical for more durable and functional prostheses. Similarly, Zubarevich et al. (2021) explored the challenges of hybrid surgery in severe calcification cases, emphasizing that personalized approaches can significantly improve clinical outcomes. Salcianu et al. (2024) furthered this research by reviewing hybrid biomaterials, underscoring their potential in regenerative applications due to their enhanced functionality and compatibility. Finally, Park et al. (2024) integrated smart sensors into hybrid valves, enabling real-time monitoring and rapid interventions in case of dysfunctions. These advances suggest a future where hybrid valves may not only offer greater durability and safety but also revolutionize the follow-up and personalization of treatment for patients with complex heart conditions.

As innovations in valve design continue to evolve, the next frontier lies in the incorporation of smart technologies. Intelligent heart valves with embedded sensors bring real-time monitoring to the forefront, allowing for precise tracking of valve function and early detection of potential complications. Bailoor et al. developed a novel, non-invasive, and non-toxic valve monitoring technique for transcatheter aortic valves (TAVs) using microsensors applied in valve stents which could measure pressure changes in prosthesis. The data was collected from critical points (Aortic sinus, Sino-tubular junction), analyzed by supervised algorithms, and demonstrated to achieve 90% accuracy in detecting compromised leaflet anomaly (Bailoor et al., 2022). Similarly, Gironi et al. used sensors to detect valve leaflet dynamics changes by intravalvular impedance sensing (IVI) method in bioprostheses. It was shown that the positioning of the electrodes influences directly IVI signal, where electrodes positioned in the commissures showed more sensibility when compared to those positioned onto the stent (Gironi et al., 2022).

The SavvyWire (OpSens Medical) is a support wire for TAVR with a distal pressure sensor (Farjat-Pasos et al., 2024). Farjat-Pasos et al. analyzed its safety, efficacy, and functionality in transcatheter aortic valve replacement. The results are very promising, as the authors achieved a success rate of 100% for this TAVR system. In addition, the devices demonstrated resistance and good functionality after implantation, being reported as better or similar to other TAVR support wires. The sensors-integrated valves are a promising cost-effective, and non-invasive alternative for improvements in diagnosing prosthetic valve dysfunctions, ensuring better outcomes for patients. Future research aims to combine these insights with additional techniques to expand diagnostic methods for prosthetic heart valves (Farjat-Pasos et al., 2024).

Ongoing clinical trials are investigating new technologies and long-term data on transcatheter aortic valve replacement (TAVR), focus on improving clinical outcomes, expanding indications, and evaluating the long-term durability and safety of these valves. MANTRA post-market clinical follow-up study (NCT05002543) is recruiting participants to analyze the safety and performance of the CORCYM devices and accessories used for valvular diseases (Meuris et al., 2023). They currently have three sub-studies planned, the MANTRA - Aortic Sub-Study, MANTRA - Mitral/Tricuspid Sub-Study (excluding Memo 4D) and MANTRA - Memo 4D Sub-Study. This study expects to involve approximately 2,150 subjects worldwide, and follow-up 30 days after implantation and annually, for 10 years. The primary outcome measure is scheduled for August 2025. Another active clinical trial, but not recruiting, the EARLY TAVR study (NCT03042104) assesses the safety and effectiveness of the SAPIEN three and SAPIEN 3 Ultra THVs compared to clinical surveillance (CS) in asymptomatic patients suffering from severe, calcific aortic stenosis. TRISCEND II Pivotal Trial clinical study (NCT04482062) evaluates Edwards EVOQUE tricuspid valve replacement system (with optimal medical therapy) for severe regurgitation. The completion date is estimated for December 2029, but the primary 30 days results showed at least a reduction of tricuspid regurgitation in 98%, despite major adverse events occurring (26.8%, with one death), the authors conclude that the procedure was safe, with TR reduction, and symptomatic improvement confirming the viability of the technique (Kodali et al., 2022).

Finally, the field of biofabricated and humanized animal-grown valve replacements offers intriguing insights. Despite various tissue-engineering approaches, the application of tissue-engineering matrices (TEM) to create TEHV comprises a promising alternative to heart valve replacement (Fioretta et al., 2021; Assunção et al., 2020). The TEM-based TEHV can be developed by *in vitro* or *in situ* grown, cultivating cells (allogenic or autologous cells) in scaffolds, generally produced with bioresorbable polymers. In situ-grown uses cell proliferation to induce ECM deposition on the scaffold, followed by decellularization to obtain the cell-based TEM (Fioretta et al., 2021). Motta et al. (Motta et al., 2019) developed a TEM human cell-derived (hTEM) to apply as tissue-engineered sinus valves (hTESVs), endowed with Valsalva sinuses for pulmonary valve replacement. Similarly, Lintas et al. demonstrated a human cell-derived tissue-engineered heart valves (TEHVs) for aortic valve replacement in an ovine model with a transcatheter aortic valve replacement (TAVR) system. Both works showed that tissue-engineered valves possess regenerative and remodeling abilities. These findings can help develop next-generation TAVR prostheses, bypassing the actual barriers of limited self-repair and remodeling capacities from heart valve prostheses (Motta et al., 2019; Lintas et al., 2018).

## 9 Conclusion

The prosthetic heart valve repair and replacement have been related since the 1950s and, since then, the improvement of the mechanical and bioprosthetic valves over time. Knowledge about the structure, morphology and properties of native heart valves is relevant to understanding the process of valve diseases and to choosing the prosthetic valve to serve as a substitute. The choice of mechanical or biological prosthesis will depend on each patient case. Although both show potential advantages, their disadvantages represent limitations that require further investigation.

Since their inception in the 1950s, prosthetic heart valves have undergone significant advancements, yet their limitations necessitate continued innovation. Understanding the complex structure and function of native heart valves is essential for improving current prostheses and addressing valvular diseases effectively.

The exploration of biomaterials, which prevent mechanical valves from causing thrombogenicity, is a challenge, although polymers emerge as an excellent alternative for these applications. Polymeric materials are promising due to their diverse application possibilities, which range from the production of scaffolds for tissue engineering approaches to heart valve development applications, that require further exploration to build artificial polymeric valves with anisotropic properties resembling native valves.

The degeneration of bioprosthetic valves due to calcification must be reverted to improve patients’ life expectancy; however, improvements remain limited. The major challenge is the degradation of bioprosthetic valves due to calcification, which underscores the need for advanced materials and functionalization techniques that promote cell adhesion, growth, and tissue remodeling.

Functionalization configures a potential alternative to solve the limitations of biological prostheses since delivering bioactive molecules may remodel tissues and provide cell adhesion, growth, migration, and differentiation. The prospect that 3D bioprinting can produce customized artificial valves with mechanical properties suitable to withstand dynamic deformations under biological conditions, as well as genetically modified pig xenografts, are promising alternatives to address the current limitations of artificial valves.

Emerging technologies, such as 3D bioprinting, hold the potential to produce custom-made valves with mechanical properties tailored for dynamic biological environments. Similarly, genetically modified pig xenografts show promise in addressing both immune rejection and structural deterioration. Meanwhile, hybrid designs that combine the durability of mechanical valves with the biocompatibility of biological ones offer a potential pathway to overcome long-standing limitations.

Currently, despite advances in materials science, only mechanical and bioprosthetic valves are used as substitutes in clinical medicine. Looking ahead, integrating artificial intelligence into valvular diagnostics and utilizing sensor-equipped prostheses for real-time monitoring could revolutionize patient outcomes.

In general, developing alternative cardiac valve prostheses to overcome problems such as long-term stability and biocompatibility is important. Hybrid valves, combining materials from mechanical and bioprosthesis try to solve those problems. Tissue engineering matrices are revealed to be able to produce TEHV with self-repair and remodeling. As research progresses, these advancements are expected to pave the way for next-generation heart valves that provide enhanced durability, functionality, and compatibility, ultimately transforming the landscape of cardiac care.
